# Robustness of Oscillatory Behavior in Correlated Networks

**DOI:** 10.1371/journal.pone.0123722

**Published:** 2015-04-20

**Authors:** Takeyuki Sasai, Kai Morino, Gouhei Tanaka, Juan A. Almendral, Kazuyuki Aihara

**Affiliations:** 1 Graduate School of Information Science and Technology, The University of Tokyo, Tokyo 113-8656, Japan; 2 Graduate School of Engineering, The University of Tokyo, Tokyo 113-8656, Japan; 3 Institute of Industrial Science, The University of Tokyo, Tokyo 153-8505, Japan; 4 Complex Systems Group, Universidad Rey Juan Carlos, 28933 Móstoles, Madrid, Spain; 5 Center for Biomedical Technology, Univ. Politecnica de Madrid, 28223 Pozuelo de Alarcon, Madrid, Spain; University of Maribor, SLOVENIA

## Abstract

Understanding network robustness against failures of network units is useful for preventing large-scale breakdowns and damages in real-world networked systems. The tolerance of networked systems whose functions are maintained by collective dynamical behavior of the network units has recently been analyzed in the framework called dynamical robustness of complex networks. The effect of network structure on the dynamical robustness has been examined with various types of network topology, but the role of network assortativity, or degree–degree correlations, is still unclear. Here we study the dynamical robustness of correlated (assortative and disassortative) networks consisting of diffusively coupled oscillators. Numerical analyses for the correlated networks with Poisson and power-law degree distributions show that network assortativity enhances the dynamical robustness of the oscillator networks but the impact of network disassortativity depends on the detailed network connectivity. Furthermore, we theoretically analyze the dynamical robustness of correlated bimodal networks with two-peak degree distributions and show the positive impact of the network assortativity.

## Introduction

From its beginnings, network robustness has been one of the central issues in complex network theory [1–4]. Since networked systems rely on interactions of the network units, failures of the network units and/or their interactions can lead to a large-scale breakdown in the entire network. For instance, power-line accidents in power grids can cause large-scale blackouts; cell necrosis in biological networks can induce disorders in living things; corporate failures in business networks can trigger a chain of bankruptcies. To get an insight into how to prevent such enormous damages on a widespread scale in the real-world networked systems, theoretical frameworks for understanding network robustness and vulnerability have been developed together with the advances in network science. The *structural robustness* indicates the failure tolerance of the network’s connectivity evaluated by the giant component, i.e., the size of the largest connected component. This framework has been applied to networks consisting of static nodes [1, 2]. On the other hand, the *dynamical robustness* focuses on the failure tolerance of dynamical behavior on networks where dynamical processes play important roles in their functions [5, 6]. While the structural robustness depends only on the network structure, the dynamical robustness is governed by the interplay between network structure and dynamics. Therefore, the important nodes which should be preferentially protected from failures and attacks in terms of dynamical robustness can be different from those in terms of structural robustness for the networks with the same topology [6].

So far, many studies on network robustness have focused on the effect of network structure characterized by a degree distribution, i.e., the probability distribution of the number of edges per node over the whole network. The structural and dynamical robustness of complex networks has been studied in comparison between homogeneously and heterogeneously connected networks with different forms of degree distributions [6, 7]. However, the degree distribution does not uniquely specify the network topology. Namely, networks with the same degree distribution can have different kinds of network topology. Such a difference can be measured by *network assortativity* with respect to node degrees (or degree-degree correlations) [8], the clustering coefficient [9], and other network characteristics [2]. Here we focus on the network assortativity. The network assortativity indicates how the degree of a node is correlated with the degrees of its neighboring nodes. In assortative (positively correlated) networks nodes tend to connect with nodes with similar degrees, whereas in disassortative (negatively correlated) networks high-degree nodes are more likely connected with low-degree nodes. The network assortativity is measured by the assortativity coefficient *r* which is defined as a Pearson correlation coefficient with respect to the degrees between the pair of nodes linked each other [8]: *r* > 0 for assortative networks; *r* = 0 for uncorrelated networks; *r* < 0 for disassortative networks. In general, sociological networks are assortative but technological and biological networks are disassortative [10]. The effect of the network assortativity has been widely studied from various aspects such as the percolation threshold [10–13], the epidemic threshold [14, 15], synchronization [16], and network observability [17], but little is known about its influence on the dynamical robustness of complex networks.

In the present study, we investigate the impact of the network assortativity on the dynamical robustness of coupled oscillator networks against deterioration of the oscillator units. The coupled oscillator networks have often been used as a simplified model of interacting units exhibiting oscillatory dynamics, as found in various phenomena such as circadian rhythms [18, 19], repetitive neuronal firings [20], oscillating gene expressions [21], chemical waves [22], Josephson junction arrays [23], and power grids [24]. All these studies show that collective phenomena, such as synchronization in coupled oscillator networks, are affected by the interplay between the properties of individual oscillators and the network structure [25]. The framework for studying robustness of coupled oscillator networks has been first proposed for a globally coupled network by Daido and Nakanishi [5] and subsequently applied to other globally coupled networks [26, 27], locally coupled networks [28], multilayer networks [29], and complex networks [6, 30, 31]. Recently, more extensive studies have been carried out on the recovery strategy of damaged oscillator networks [32], the dynamical robustness of coupled heterogeneous oscillators [33, 34], and the role of time delay in the dynamical robustness of oscillator networks [35]. Based on the framework used in these previous studies, we examine herewith the dynamical robustness of correlated oscillator networks. In this framework, the normal nodes are called *active* oscillators and the deteriorated ones are called *inactive* oscillators. When all the oscillator nodes are active in the normal state, we observe global oscillatory behavior in the entire network. As the fraction *p* of the inactive oscillator nodes increases, the global oscillatory behavior is weakened. At a critical fraction *p* = *p*_*c*_, the global oscillation vanishes and a non-oscillatory equilibrium state becomes stable due to a phase transition (called aging transition [5]). The critical value *p*_*c*_ is used as a measure for the dynamical robustness: a larger value of *p*_*c*_ implies a more robust network.

To consider the effect of the network assortativity, we fix the degree distribution of a given network and change the assortativity coefficient *r* by two edge-rewiring methods [8, 36]. Notice that the degree mixing of a network cannot be completely described by *r*, since it is a global measure, thus considering the two different edge-rewiring methods lets us study different joint degree distributions regardless they might have the same assortativity coefficient *r*. In numerical simulations for networks with Poisson and scale-free degree distributions, we show that in most cases the network assortativity enhances the dynamical robustness of oscillator networks. The results also indicate that the effect of the network disassortativity on the dynamical robustness is different between the network reshuffling methods. We demonstrate that this difference is caused by the fact that disassortative networks generated by the two edge-rewiring methods have essentially different types of network topology even if they have the same assortativity coefficients. In order to theoretically approach the dynamical robustness of correlated networks, we focus on the extreme cases with bimodal oscillator networks where the degrees of the oscillator nodes are limited to two values. The critical fraction *p*_*c*_ is analytically derived and numerically validated for the correlated bimodal networks. The results indicate the positive role of assortativity in the dynamical robustness of oscillator networks. We conclude that the network assortativity is beneficial for the dynamical robustness of coupled oscillator networks. This is consistent with the conclusions from the analysis of the structural robustness of correlated networks [37].

## Methods

### Dynamical robustness of coupled oscillator networks

**Fig 1:**
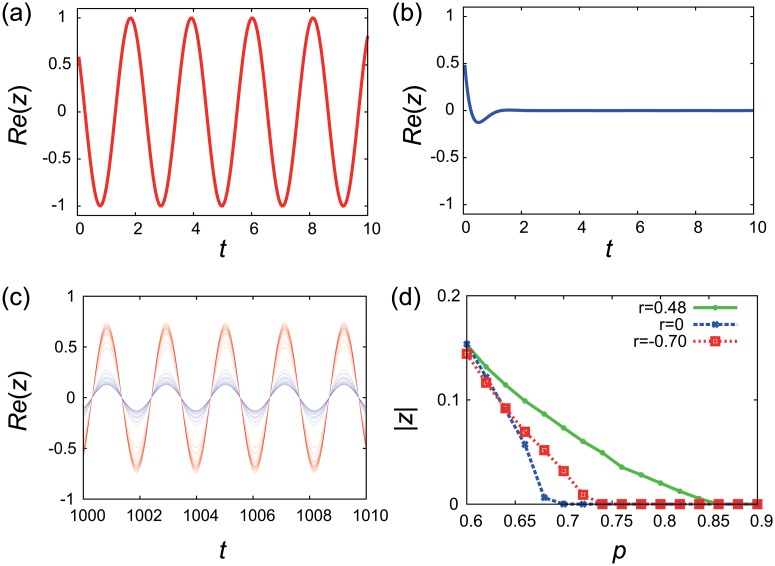
Dynamical robustness of coupled SL oscillator networks. (a) Limit-cycle oscillation produced by the single isolated active oscillator. (b) Damping oscillation produced by the single isolated inactive oscillator. (c) The global oscillatory behavior in an uncorrelated network of active and inactive oscillators. The time evolutions of the state variables of some active (red) and inactive (blue) oscillator nodes are plotted. The parameters are set at *N* = 3000, *p* = 0.4, and *r* = 0. (d) The order parameter |*Z*| plotted against the fraction *p* of the inactive oscillators in uncorrelated (*r* = 0), assortative (*r* = 0.48), and disassortative (*r* = −0.7) networks with power-law degree distributions. The correlated networks were generated by the GER method.

We examine the effect of the network assortativity on the dynamical robustness of coupled oscillator networks [5, 6, 34]. Each oscillator is represented by the Stuart-Landau (SL) oscillator [22]. The SL oscillator is equivalent to the normal form of the supercritical Hopf bifurcation, which is a typical mechanism for the onset of oscillatory behavior in dynamical systems [38]. By adjusting the control parameter responsible for the supercritical Hopf bifurcation, the single SL oscillator can be either active or inactive. The network model consisting of *N* diffusively coupled SL oscillators is described as follows [5, 6]:

$$\begin{array}{ccc} {\dot{z}}_{j} & = & (\alpha_{j}+i\Omega -{\left|z_{j}\right|}^{2})z_{j}+\frac{K}{N}\sum_{k=1}^{N}a_{jk}(z_{k}-z_{j})\;\text{for}\;j=1,\ldots ,N, \end{array}$$ (1)

where *i* stands for the imaginary unit, *z*_*j*_ ∈ 𝓒 is the complex state variable of oscillator node *j*, *α*_*j*_ ∈ 𝓡 is the control parameter of oscillator node *j*, Ω ∈ 𝓡 is the natural frequency, *K* ∈ 𝓡 is the coupling strength, and *a*_*jk*_ ∈ {0,1} is the (*j*, *k*) entry of the adjacency matrix *A* = (*a*_*jk*_) characterizing the network connectivity. We set *a*_*jk*_ = 1 if the connection is present between node *j* and node *k*, and *a*_*jk*_ = 0 otherwise. We assume that the connections are bidirectional, i.e., *a*_*jk*_ = *a*_*kj*_ for any *j* and *k*, and there are no self-connections, i.e., *a*_*jj*_ = 0 for *j* = 1,…, *N*. The degree of oscillator node *j* is given by $\sum_{k=1}^{N}a_{jk}$ and the link density is denoted by $d\equiv \sum_{j=1}^{N}\sum_{k=1}^{N}a_{jk}/(N(N-1))$. When the oscillator is isolated (Eq (1) with *K* = 0), the active oscillator with *α*_*j*_ = *a* > 0 exhibits self-sustained limit-cycle oscillation (Fig 1(a)) and the inactive oscillator with *α*_*j*_ = −*b* < 0 settles into a quiescent state after damping oscillation (Fig 1(b)) [5, 6]. The parameters *a* and *b* are the positive real values. Note that the inactive oscillator is not able to oscillate when isolated but can exhibit oscillation through coupling with the neighboring active oscillators in the network as shown in Fig 1(c). Throughout this paper, the parameters are set at *N* = 3000, *K* = 30, *d* = 0.08, *a* = 1, and *b* = 3, unless otherwise noted.

Whether the global oscillatory behavior is observed or not depends on various factors such as the network topology, the fraction *p* of the inactive oscillators, and the configuration of the active and inactive oscillators. Fig 1(d) shows the decay of the global oscillatory behavior with an increase in *p* for uncorrelated, assortative, and disassortative networks. The strength of the global oscillatory behavior in the entire network is measured by the order parameter |*Z*(*t*)| where $Z(t)\equiv \sum_{j=1}^{N}z_{j}(t)/N$. We numerically integrate the coupled oscillator model (Eq 1) using the fourth-order Runge-Kutta method with time step 0.05 and calculate the order parameter |*Z*(*t*)| at *t* = 50000. Notice that, in general, the order parameter fluctuates in time and its measure requires a temporal average, but our simulations show that this time period is long enough for the order parameter to approximately converge to a steady-state value. As *p* increases from 0, the order parameter declines gradually and vanishes at a critical fraction *p*_*c*_. We consider that the global oscillation has stopped if the order parameter decreases to 10^−6^ in numerical simulations. The critical fraction *p*_*c*_ is employed as a measure for the dynamical robustness of oscillator networks, i.e., a larger value of *p*_*c*_ implies that the network is more failure tolerant. We find that the decline curves of the order parameter are different depending on the assortativity coefficient *r*, yielding different values of *p*_*c*_. In terms of the critical value *p*_*c*_, the assortative network seems to be more robust compared with the uncorrelated and disassortative networks. We investigate how the critical value *p*_*c*_ depends on the network assortativity in the Results section.

### Network assortativity

The assortativity coefficient *r* is an index to measure the network assortativity, or the degree-degree correlations [8], which is defined as the Pearson correlation coefficient of the degrees between pairs of connected nodes. To define the assortativity coefficient, we denote the degree distribution of a network by *P*(*k*), which is the probability that a randomly chosen node has degree *k*. Let us consider the probability that a node in the end of a randomly chosen edge has *k* edges except for the chosen one. Such a number of edges, which is one less than the total degree, is called the *remaining degree*[8]. A node with remaining degree *k* has degree *k* + 1 totally and the probability that an edge leaving such a node is chosen is proportional to *k* + 1. Therefore, the probability distribution of the remaining degree is proportional to (*k* + 1)*P*(*k* + 1). The normalized distribution of the remaining degree of the node at the end of a randomly chosen edge is given by

$$\begin{array}{ccc} Q(k) & = & \frac{\left(k+1)P(k+1\right)}{\sum_{j}\;jP\left(j\right)}. \end{array}$$ (2)

Now we consider the joint probability distribution *E*(*j*, *k*) of the remaining degrees of the two nodes (one with degree *j* and the other with degree *k*) at either end of a randomly chosen edge [39]. This quantity satisfies *E*(*j*, *k*) = *E*(*k*, *j*), ∑_*j*_∑_*k*_ *E*(*j*, *k*) = 1, and ∑_*j*_ *E*(*j*, *k*) = *Q*(*k*). The level of assortativity is quantified by the correlation function with respect to node degrees as ⟨*jk*⟩−⟨*j*⟩⟨*k*⟩ = ∑_*j*_∑_*k*_ *jk*(*E*(*j*, *k*)−*Q*(*j*)*Q*(*k*)), where the brackets indicate an average over edges. For uncorrelated networks, the remaining degrees are independent, i.e., *E*(*j*, *k*) = *Q*(*j*)*Q*(*k*), and therefore, the assortativity level is 0. By normalizing the correlation function with its maximal value achieved when *E*(*j*, *k*) = *Q*(*k*)*δ*_*jk*_, the assortativity coefficient *r* is defined as follows:

$$\begin{array}{ccc} r & \equiv & \frac{1}{\sigma_{q}^{2}}\sum_{j}\sum_{k}jk\left(E\left(j,k\right)-Q\left(j\right)Q\left(k\right)\right), \end{array}$$ (3)

where the normalizing factor is the variance of the distribution *Q*(*k*), i.e., $\sigma_{q}^{2}\equiv \sum_{k}k^{2}Q(k)-{\left(\sum_{k}kQ(k)\right)}^{2}$. The range of *r* is −1 ≤ *r* ≤ 1: *r* > 0 for assortative networks, *r* = 0 for uncorrelated networks, and *r* < 0 for disassortative networks. The assortativity coefficient *r* in Eq (3) can be rewritten as follows:

$$\begin{array}{ccc} r & = & \frac{4M\sum_{m}j_{m}k_{m}-{\left[\sum_{m}(j_{m}+k_{m})\right]}^{2}}{2M\sum_{m}(j_{m}^{2}+k_{m}^{2})-{\left[\sum_{m}(j_{m}+k_{m})\right]}^{2}}, \end{array}$$ (4)

where *M* is the total number of edges, *m* ∈ {1,…, *M*} is the index of edges, and *j*_*m*_ and *k*_*m*_ represent the degrees of the two nodes connected by edge *m* [8].

In our numerical simulations, we change the assortativity coefficient *r* using two edge-rewiring methods: one is proposed by Xulvi-Brunet and Sokolov [36] and the other by Newman [8]. We call the former method the greedy edge rewiring (GER) and the latter method the stochastic edge rewiring (SER). We start with an uncorrelated network with *r* ≈ 0 and reshuffle the network edges without allowing self-loops and overlaps. We choose two edges randomly from the network, represented by the connected node pairs, (*v*_1_, *w*_1_) and (*v*_2_, *w*_2_). The remaining degrees of these node pairs are correspondingly denoted by (*j*_1_, *k*_1_) and (*j*_2_, *k*_2_). We rewire the edges to control the network assortativity. The rewiring process does not alter the number of edges for each node, and hence, the degree distribution is kept unchanged.

**Fig 2:**
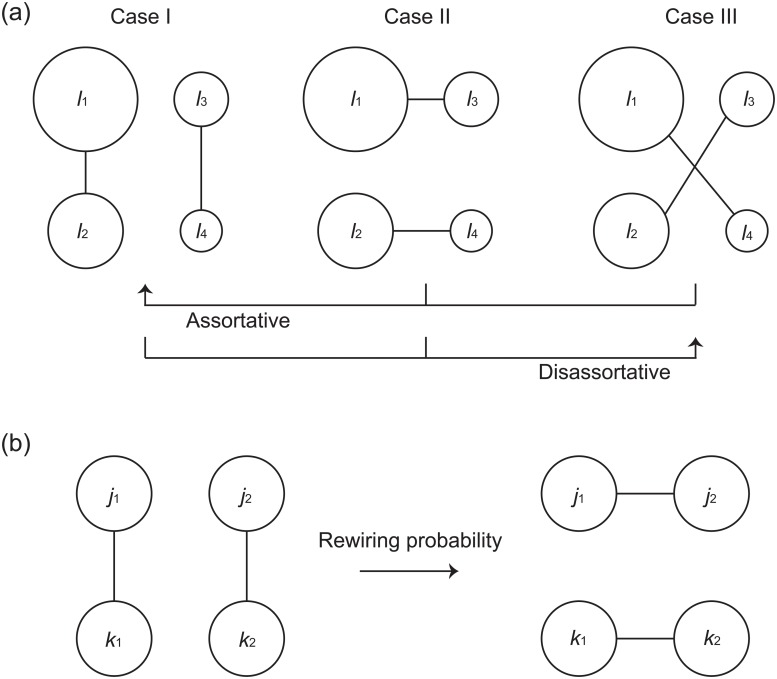
Network reshuffling methods for changing the network assortativity. (a) The greedy edge-rewiring (GER) method [36]. The remaining degrees of the two connected node pairs, (*j*_1_, *k*_1_) and (*j*_2_, *k*_2_), are sorted in the descending order and relabeled as *l*_1_, *l*_2_, *l*_3_, and *l*_4_ so that *l*_1_ ≥ *l*_2_ ≥ *l*_3_ ≥ *l*_4_. The size of the node corresponds to its remaining degree. When making the network assortative, Case I is chosen if the current state is Case II or III. When making the network disassortative, Case III is chosen if the current state is Case I or II. (b) The stochastic edge-rewiring (SER) method [8]. The acceptance probability for edge rewiring is given by $\text{min}\left\{1,\frac{E(j_{1},j_{2})E(k_{1},k_{2})}{E(j_{1},k_{1})E(j_{2},k_{2})}\right\}$ where *E*(*j*, *k*) is the joint probability distribution for the remaining degrees of the two nodes in the end of a randomly chosen edge.

In the GER method, the edge rewiring is conducted based on the degrees of the connected node pairs. We sort the remaining degrees *j*_1_, *j*_2_, *k*_1_, and *k*_2_ in descending order and relabel them to *l*_1_, *l*_2_, *l*_3_ and *l*_4_ so that *l*_1_ ≥ *l*_2_ ≥ *l*_3_ ≥ *l*_4_. Fig 2(a) illustrates the three possibilities for separating the four nodes into two pairs of connected nodes. When increasing the assortativity coefficient, Case I is chosen to set the edges between the nodes with more similar degrees if the current state is Case II or III. When decreasing the assortativity coefficient, Case III is chosen to set an edge between the nodes with the largest and smallest degrees if the current state is Case I or II. We increase or decrease the assortativity coefficient by repeating the edge rewiring in a greedy way and continue until the assortativity coefficient is no longer changed. The assortativity coefficient *r* monotonically increases or decreases with this method.

In the SER method, we repeat the edge rewiring stochastically to change the network assortativity, inspired by the Metropolis dynamics introduced by Newman [8]. Newman used a numerical method to generate a network satisfying a given joint probability distribution *E*(*j*, *k*) of the remaining degrees, because it is not trivial to find such a network due to topological constraints. In this method, the chosen node pairs, (*v*_1_, *w*_1_) and (*v*_2_, *w*_2_), are replaced by the new ones, (*v*_1_, *v*_2_) and (*w*_1_, *w*_2_), with acceptance probability $\text{min}\left\{1,\frac{E(j_{1},j_{2})E(k_{1},k_{2})}{E(j_{1},k_{1})E(j_{2},k_{2})}\right\}$ as illustrated in Fig 2(b). In our study, we employ the following symmetric binomial form [8]:

$$\begin{array}{ccc} E(j,k) & = & 𝓝e^{-(j+k)/\kappa }\left(C\left(j+k,j\right)f^{j}g^{k}+C\left(j+k,k\right)f^{k}g^{j}\right), \end{array}$$ (5)

where *C*(*m*, *n*) ≡ *m*!/(*n*!(*m* − *n*)!), *f* + *g* = 1, *κ* > 0, and 𝓝 (1 − *e* ^−1/*κ*^)/2 is a normalization factor. Note that our aim of edge rewiring is not to achieve the above *E*(*j*, *k*) but to change the network assortativity. The probability *f* is the control parameter for the assortativity coefficient. In numerical simulations, we set *f* = 0.5 when increasing the assortativity and *f* = 0.05 when decreasing the assortativity [8]. The value of *κ* is set at *κ* = 100.

## Results

### Dynamical robustness of correlated complex networks

#### Correlated networks with Poisson degree distributions

First, we examine the dynamical robustness of oscillator networks with Poisson degree distributions. The uncorrelated network is initially given as an Erdős-Rényi random graph [40], where the degrees are concentrated around the mean degree. Then the network is changed to be assortative or disassortative using the edge-rewiring techniques introduced in the Methods section. Since each rewiring method modifies the assortativity coefficient *r* operating differently on the joint degree distribution, the reachable ranges of *r* are not the same. The SER method is a random process and, consequently, extreme values of *r* are unlikely to be reached. On the contrary, the GER method is a greedy target-oriented method that is able to find those specific networks, even though they are a rather small subset of all possible networks having the same degree distribution. Next, for each network generated by the edge-rewiring methods, we increase the fraction *p* of inactive oscillators from 0 until we find the critical value *p*_*c*_ at which the order parameter vanishes. The critical value *p*_*c*_ depends on the order in which the oscillators are inactivated with an increase in *p*. We consider two ways of oscillator inactivation [6]: *random* inactivation where the inactive oscillators are randomly chosen; *targeted* inactivation where the oscillator nodes are inactivated in the order (or the inverse order) of the degree.

**Fig 3:**
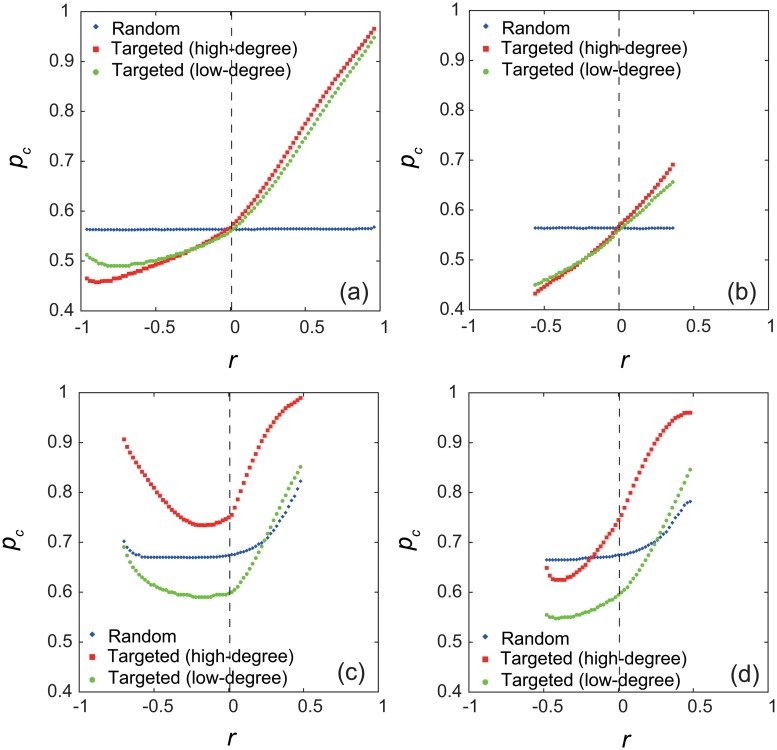
The critical value *p*_*c*_ for correlated networks. In each panel, the numerically obtained values of the critical fraction *p*_*c*_ are plotted against the assortativity coefficient *r* for random and targeted inactivation. The system size is *N* = 3000. The uncorrelated network with *r* ≈ 0 is given by the Erdős-Rényi random graph [40] in (a) and (b) and by the BA scale-free network [41] in (c) and (d). (a) Networks with Poisson degree distributions, generated by the GER method. (b) Networks with Poisson degree distributions, generated by the SER method. (c) Networks with power-law degree distributions, generated by the GER method. (d) Networks with power-law degree distributions, generated by the SER method.

Figs 3(a) and 3(b) show the critical value *p*_*c*_ for the networks generated by the GER and SER methods, respectively. For uncorrelated networks with *r* ≈ 0, the value of *p*_*c*_ is the same for the three types of inactivation, because the way of oscillator inactivation is not significant in the homogeneously connected random network [6]. For the random inactivation in both panels, the value of *p*_*c*_ is almost constant, independently of the *r* value. This is because the number of inactive oscillators in the neighborhood of each oscillator node is not affected by *r*. In fact, the oscillation amplitudes of the individual oscillators have similar distributions for disassortative, uncorrelated, and assortative networks (S1 and S2 Fig).

For the targeted inactivation of high-degree oscillator nodes and that of low-degree oscillator nodes, the value of *p*_*c*_ monotonically increases with *r* as shown in Figs 3(a) and 3(b). This result is attributed to the property that the amplitudes of the active oscillators, dominantly contributing to the order parameter, are larger for more assortative networks (S1 and S2 Fig). We can conclude that the network assortativity has a positive effect on the dynamical robustness of oscillator networks against targeted inactivation.

#### Correlated networks with power-law degree distributions

Second, we performed similar numerical experiments using correlated scale-free networks with power-law degree distributions. The initial uncorrelated network is given by a Barabási-Albert (BA) scale-free network [41], which consists of a small number of highly connected nodes (hubs) and a large number of loosely connected nodes.

Figs 3(c) and 3(d) show the critical fraction *p*_*c*_ plotted against *r* for the GER and SER methods, respectively. For uncorrelated networks with *r* ≈ 0, the value of *p*_*c*_ for targeted inactivation of low-degree nodes is smaller than the values for the other two inactivation types. This is consistent with the previous study showing the crucial role of low-degree nodes for the dynamical robustness [6]. However, when *r* is varied, the curves of the *p*_*c*_ values make intersections as shown in Figs 3(c) and 3(d), indicating that the important nodes for the dynamical robustness can change depending on the network assortativity. For all the inactivation types, the value of *p*_*c*_ monotonically increases as *r* is increased from 0 as shown in Figs 3(c) and 3(d). It is shown that the oscillation amplitudes of the active oscillators are smaller for the higher-degree nodes in the random inactivation case (S3 and S4 Fig). This means that the higher-degree nodes decrease their oscillation levels more to recover the oscillations of the larger number of neighboring inactive oscillator nodes.

In assortative networks, the connections tend to be made between high-degree nodes and between low-degree nodes. Therefore, for the targeted inactivation of high-degree nodes, the low-degree active oscillators connected to few inactive oscillators can maintain the large oscillation amplitudes (S3 and S4 Fig). Similarly, for the targeted inactivation of low-degree nodes, the high-degree active oscillators connected to few inactive oscillators can keep the large oscillation amplitudes (S3 and S4 Fig). These nodes maintaining the large oscillation amplitudes are responsible for the large value of *p*_*c*_, i.e., the highly robust oscillatory behavior. Thus, we confirm the positive role of assortativity in the dynamical robustness.

On the other hand, the effect of disassortativity is different between the edge-rewiring methods as shown in Figs 3(c) and 3(d). As *r* is decreased from 0, the critical value *p*_*c*_ increases after a slight downward trend for the GER method as shown in Fig 3(c), but it almost monotonically decreases until *r* ≈ −0.5 for the SER method as shown in Fig 3(d). We notice the qualitative difference in the distributions of the oscillation amplitudes, particularly for the high-degree nodes (S3 and S4 Fig). This suggests that the networks with the same negative assortativity coefficient can have essentially different types of topology. We next examine the detailed connectivity of the correlated scale-free networks to clarify the difference between the edge-rewiring methods.

#### Connectivity matrices of correlated networks

**Fig 4:**
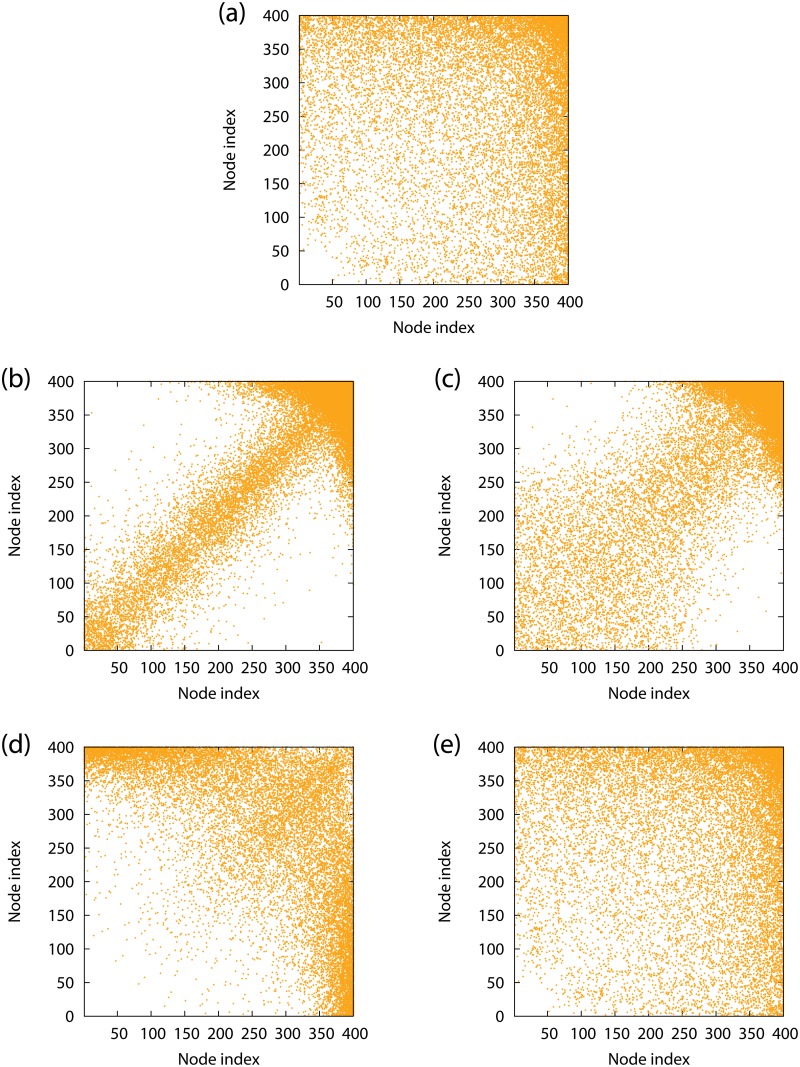
Adjacency matrices of scale-free networks. The dots located at (*i*, *j*) indicate the presence of the edges between node *i* and node *j*. (a) An uncorrelated network with *r* ≈ 0. (b) An assortative network with *r* = 0.4, generated by the GER method. (c) The same as (b), but generated by the SER method. (d) A disassortative network with *r* = −0.48, generated by the GER method. (e) The same as (d), but generated by the SER method.

The similarity and difference between the correlated scale-free networks generated by the two edge-rewiring methods are clarified in Fig 4. Fig 4(a) shows the adjacency matrix of the BA scale-free network with *r* ≈ 0 [41]. The dots are dense in the upper-right corner due to the hubs which have a large number of edges.

Figs 4(b) and 4(c) show the adjacency matrices of assortative scale-free networks with the same positive assortativity coefficient *r* obtained by the GER and SER methods, respectively. The dots are densely plotted along the diagonal line in both figures and this tendency is strengthened as *r* increases. Therefore, the monotonic increase in *p*_*c*_ with *r* is common to the networks generated by the two edge-rewiring methods. However, the variance from the diagonal line seems to be smaller for the network obtained by the GER method than that obtained by the SER method. This detailed topological difference results in the different values of *p*_*c*_ as shown in Figs 3(c) and 3(d).

**Fig 5:**
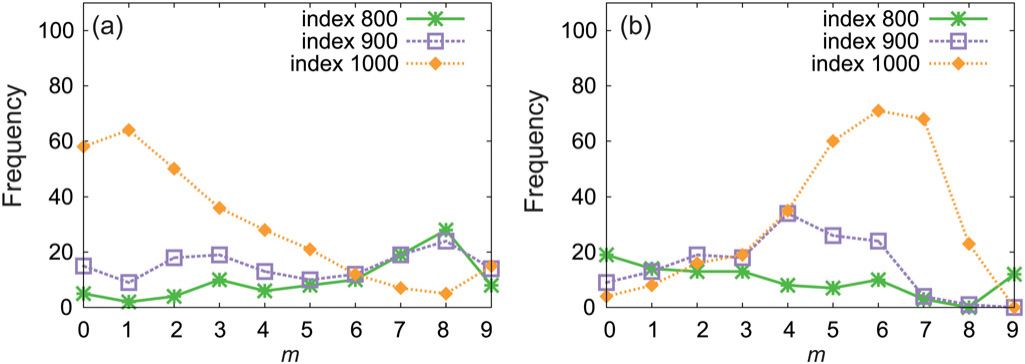
The histogram of the degrees of the neighboring nodes in the disassortative networks. The network is given by scale-free network with size *N* = 1000 and assortativity coefficient *r* = −0.48. The number of neighboring nodes with indices in the range [100*m* + 1,100(*m* + 1) + 1) (*m* = 1,…,9) is plotted for the nodes with index 800, 900, and 1000. (a) The network obtained by the GER method. (b) The network obtained by the SER method.

Figs 4(d) and 4(e) show the adjacency matrices of disassortative scale-free networks obtained by the GER and SER methods, respectively. There are many dots in the off-diagonal parts, corresponding to the connections between high-degree and low-degree nodes, in Fig 4(d), but not conspicuously in Fig 4(e). To quantify the difference in the adjacency matrices between the two disassortative networks, we investigate how the high-degree nodes with indices 800, 900, and 1000 are connected to the neighboring nodes. Figs 5(a) and 5(b) show the histograms of the number of neighboring nodes with respect to each range of index values, [100*m* + 1,100(*m* + 1) + 1) (*m* = 0,…,9), for the GER and SER methods, respectively. In Fig 5(a), the nodes with indices 800 and 900 are connected to the nodes with similar degrees but the node with index 1000 is mainly connected to the low-degree nodes. These two properties emerge as the diagonal plots and the off-diagonal plots in the adjacency matrix in Fig 4(d), respectively. Although the former property contributes to increasing the assortativity coefficient, the assortativity coefficient is negative because of the greater impact of the latter property decreasing the coefficient. On the other hand, in Fig 5(b), the three high-degree nodes with indices 800, 900, and 1000 tend to be connected to the nodes with intermediate degrees. The connections to the nodes with similar degrees are very few. Therefore, the assortativity coefficient becomes negative. These two examples of disassortative networks demonstrate that the dynamical robustness of oscillator networks depends on the detailed network structure which is not uniquely determined by the assortativity coefficient *r*.

#### Verification of the role of the network assortativity

Fig 3, which shows the dependency of the critical value *p*_*c*_ on the assortativity coefficient *r*, is computed assuming that the system size is *N* = 3000, the coupling strength is *K* = 30, and the parameters for individual oscillators are fixed at (*a*, *b*) = (1,3). Hence, it is important to determine if the features found in Fig 3 are sensitive to other parameter settings. The results for system size *N* = 1500 (S5 Fig) are almost the same as those in Fig 3, suggesting that the critical value *p*_*c*_ is almost independent of the system size. The results for coupling strength *K* = 40 (S6 Fig) are qualitatively similar to those in Fig 3, but the increase in the coupling strength reduces *p*_*c*_ as in the uncorrelated networks [6]. The results for *b* = 5 (S7 Fig) are also qualitatively similar to those in Fig 3. Consequently, we confirm that the network assortativity has a positive role in the dynamical robustness of oscillator networks for the different parameter settings.

Furthermore, we tested the case with heterogeneous oscillators [34, 42]. The parameter *α*_*j*_ in Eq (1) is randomly chosen from a uniform distribution with the range of [*μ*−*δ*/2, *μ* + *δ*/2] where *μ* is the average of *α*_*j*_ and *δ* is the distribution width corresponding to the degree of heterogeneity. As *μ* is decreased from a sufficiently large value, the global oscillation vanishes at a critical point *μ*_*c*_. Instead of *p*_*c*_, as in the case with homogeneous oscillators, now *μ*_*c*_ is used as a measure of dynamical robustness in the coupled heterogeneous oscillators [34]. The smaller the value of *μ*_*c*_ is, the more robust the oscillator network is. The results for *δ* = 2 (S8 Fig) show that the value of *μ*_*c*_ is almost unchanged for the variation of *r* in the networks with Poisson degree distributions, but in the networks with power-law degree distributions it decreases as *r* increases in the positive range. The latter result means that the network assortativity enhances the dynamical robustness of heterogeneously coupled networks of heterogeneous oscillators.

### Dynamical robustness of correlated bimodal networks

To theoretically approach the impact of network assortativity on the dynamical robustness of coupled oscillator networks, we consider an extreme case using bimodal networks [43] (or two-peak random networks [44]) where the degrees are limited to two values. We analytically derive the critical fraction *p*_*c*_ for the coupled oscillator model (Eq 1) with correlated bimodal networks. Note that the topology of the bimodal network is uniquely determined for a given value of *r*.

#### Random inactivation

First we analyze the critical fraction for the random inactivation. We employ the heterogeneous mean field approximation to derive the critical fraction *p*_*c*_ [6]. We denote the two degrees by *k*_1_ and *k*_2_ for the bimodal networks. We assume that the oscillator node with degree *k*_*m*_ (*m* = 1, 2) has *N*_*k*_*m*_*k*_*n*__ neighboring oscillator nodes with degree *k*_*n*_ (*n* = 1, 2) on average. Then, the interaction of an oscillator node with degree *k*_*m*_ with the neighboring nodes is divided into four types depending on whether the neighboring node is active or inactive and whether the degree of the neighboring node is *k*_1_ or *k*_2_. Namely, the state variables are reduced to four representative variables: *A*_*k*_*m*__ for active oscillators with degree *k*_*m*_ and *I*_*k*_*m*__ for inactive oscillators with degree *k*_*m*_ (*m* = 1, 2). Now we assume based on numerical observations that the oscillator nodes with the same activity type and the same degree behave identically. Let the degree of oscillator *j* is *k*_*m*_ (*m* = 1, 2) in Eq (1). Then the inflow term is approximated as follows:

$$\begin{array}{ccc} \sum_{k=1}^{N}a_{jk}z_{k} & \approx & q\left(N_{k_{m}k_{1}}A_{k_{1}}+N_{k_{m}k_{2}}A_{k_{2}}\right)+p\left(N_{k_{m}k_{1}}I_{k_{1}}+N_{k_{m}k_{2}}I_{k_{2}}\right), \end{array}$$ (6)

where *q* ≡ 1 − *p*. Here we define the mean fields for the active and inactive oscillators with degree *k*_*m*_ (*m* = 1, 2), respectively, as follows:

$$\begin{array}{ccc} H_{k_{m}}^{A} & \equiv & \sum_{n=1}^{2}\frac{N_{k_{m}k_{n}}}{k_{m}}A_{k_{n}}\approx \sum_{n=1}^{2}\frac{E(k_{m},k_{n})}{Q(k_{m})}A_{k_{n}}, \end{array}$$ (7)

$$\begin{array}{ccc} H_{k_{m}}^{I} & \equiv & \sum_{n=1}^{2}\frac{N_{k_{m}k_{n}}}{k_{m}}I_{k_{n}}\approx \sum_{n=1}^{2}\frac{E(k_{m},k_{n})}{Q(k_{m})}I_{k_{n}}, \end{array}$$ (8)

where the above approximations come from Eq (2). Using these expressions, the model equations in Eq (1) can be reduced for *m* = 1, 2 as follows:

$$\begin{array}{ccc} {\dot{A}}_{k_{m}} & = & (a+i\Omega -{\left|A_{k_{m}}\right|}^{2})A_{k_{m}}+\frac{Kk_{m}}{N}(qH_{k_{m}}^{A}+pH_{k_{m}}^{I}-A_{k_{m}}), \end{array}$$ (9)

$$\begin{array}{ccc} {\dot{I}}_{k_{m}} & = & (-b+i\Omega -{\left|I_{k_{m}}\right|}^{2})I_{k_{m}}+\frac{Kk_{m}}{N}(qH_{k_{m}}^{A}+pH_{k_{m}}^{I}-I_{k_{m}}). \end{array}$$ (10)

Based on numerical simulations showing that all the oscillators in the entire network exhibit phase synchronization after a transient period, we set

$$\begin{array}{cc} A_{k_{m}}\left(t\right) & =r_{k_{m}}^{A}\left(t\right)\;\exp\left(i(\Omega t+\theta )\right), \end{array}$$ (11)

$$\begin{array}{cc} I_{k_{m}}\left(t\right) & =r_{k_{m}}^{I}\left(t\right)\;\exp(i(\Omega t+\theta )), \end{array}$$ (12)

where $r_{k_{m}}^{A}$ and $r_{k_{m}}^{I}$ represent the oscillation amplitudes of the active and inactive oscillators with degree *k*_*m*_, respectively, Ω is the oscillation frequency, and *θ* is the phase delay. Substituting Eqs (11)–(12) into Eqs (7)–(8), the mean fields for the state variables are represented as follows:

$$\begin{array}{ccc} H_{k_{m}}^{A}\left(t\right) & = & R_{k_{m}}^{A}\left(t\right)\;\exp(i(\Omega t+\theta )), \end{array}$$ (13)

$$\begin{array}{ccc} H_{k_{m}}^{I}\left(t\right) & = & R_{k_{m}}^{I}\left(t\right)\;\exp(i(\Omega t+\theta )), \end{array}$$ (14)

where the mean fields for the oscillation amplitudes are written as

$$\begin{array}{ccc} R_{k_{m}}^{A}\left(t\right) & \equiv & \sum_{n=1}^{2}\frac{E(k_{m},k_{n})}{q_{k_{m}}}r_{k_{n}}^{A}\left(t\right), \end{array}$$ (15)

$$\begin{array}{ccc} R_{k_{m}}^{I}\left(t\right) & \equiv & \sum_{n=1}^{2}\frac{E(k_{m},k_{n})}{q_{k_{m}}}r_{k_{n}}^{I}\left(t\right). \end{array}$$ (16)

Substituting Eqs (11)–(12) into Eqs (9)–(10), we obtain the equations with respect to the oscillation amplitudes as follows:

$$\begin{array}{ccc} {\dot{r}}_{k_{m}}^{A} & = & (a-\frac{Kk_{m}}{N}-{\left(r_{k_{m}}^{A}\right)}^{2})r_{k_{m}}^{A}+\frac{Kk_{m}}{N}(qR_{k_{m}}^{A}+pR_{k_{m}}^{I}), \end{array}$$ (17)

$$\begin{array}{ccc} {\dot{r}}_{k_{m}}^{I} & = & (-b-\frac{Kk_{m}}{N}-{\left(r_{k_{m}}^{I}\right)}^{2})r_{k_{m}}^{I}+\frac{Kk_{m}}{N}(qR_{k_{m}}^{A}+pR_{k_{m}}^{I}). \end{array}$$ (18)

Now let us assume that $R_{k_{m}}^{A}$ and $R_{k_{m}}^{I}$ are given for *m* = 1, 2. The oscillation amplitudes for the steady-state oscillations are calculated as the positive real roots of the following cubic equations:

$$\begin{array}{ccc} {\left(r_{k_{m}}^{A}\right)}^{3}-(a-\frac{Kk_{m}}{N})r_{k_{m}}^{A}-\frac{Kk_{m}}{N}(qR_{k_{m}}^{A}+pR_{k_{m}}^{I}) & = & 0, \end{array}$$ (19)

$$\begin{array}{ccc} {\left(r_{k_{m}}^{I}\right)}^{3}-(-b-\frac{Kk_{m}}{N})r_{k_{m}}^{I}-\frac{Kk_{m}}{N}(qR_{k_{m}}^{A}+pR_{k_{m}}^{I}) & = & 0. \end{array}$$ (20)

For these equations to have real roots, we assume *k*_min_ > *aN*/*K* where *k*_min_ ≡ min(*k*_1_, *k*_2_) [6, 34]. By solving the cubic equations, the oscillation amplitudes are represented as $r_{k_{m}}^{A*}(R_{k_{m}}^{A},R_{k_{m}}^{I})$ and $r_{k_{m}}^{I*}(R_{k_{m}}^{A},R_{k_{m}}^{I})$, respectively (see Refs. [6, 34] for the detailed form of the solutions). The mean fields of the oscillation amplitudes should be reconstructed from Eqs (15)–(16) using these steady-state solutions. Hence, the self-consistent condition is represented as follows:

$$\begin{array}{ccc} R_{k_{m}}^{A} & = & G_{k_{m}}^{A}(R_{k_{1}}^{A},R_{k_{2}}^{A},R_{k_{1}}^{I},R_{k_{2}}^{I}), \end{array}$$ (21)

$$\begin{array}{ccc} R_{k_{m}}^{I} & = & G_{k_{m}}^{I}(R_{k_{1}}^{A},R_{k_{2}}^{A},R_{k_{1}}^{I},R_{k_{2}}^{I}), \end{array}$$ (22)

for *m* = 1, 2, where

$$\begin{array}{ccc} G_{k_{m}}^{A}(R_{k_{1}}^{A},R_{k_{2}}^{A},R_{k_{1}}^{I},R_{k_{2}}^{I}) & = & \sum_{n=1}^{2}\frac{E(k_{m},k_{n})}{Q(k_{m})}r_{k_{n}}^{A*}(R_{k_{n}}^{A},R_{k_{n}}^{I}), \end{array}$$ (23)

$$\begin{array}{ccc} G_{k_{m}}^{I}(R_{k_{1}}^{A},R_{k_{2}}^{A},R_{k_{1}}^{I},R_{k_{2}}^{I}) & = & \sum_{n=1}^{2}\frac{E(k_{m},k_{n})}{Q(k_{m})}r_{k_{n}}^{I*}(R_{k_{n}}^{A},R_{k_{n}}^{I}). \end{array}$$ (24)

The linearized matrix evaluated at the steady-state equilibrium is given by

$$\begin{array}{ccc} J_{0} & = & {(\begin{array}{cccc} \frac{\partial G_{k_{1}}^{A}}{\partial R_{k_{1}}^{A}} & \frac{\partial G_{k_{1}}^{A}}{\partial R_{k_{2}}^{A}} & \frac{\partial G_{k_{1}}^{A}}{\partial R_{k_{1}}^{I}} & \frac{\partial G_{k_{1}}^{A}}{\partial R_{k_{2}}^{I}}\\ \frac{\partial G_{k_{2}}^{A}}{\partial R_{k_{1}}^{A}} & \frac{\partial G_{k_{2}}^{A}}{\partial R_{k_{2}}^{A}} & \frac{\partial G_{k_{2}}^{A}}{\partial R_{k_{1}}^{I}} & \frac{\partial G_{k_{2}}^{A}}{\partial R_{k_{2}}^{I}}\\ \frac{\partial G_{k_{1}}^{I}}{\partial R_{k_{1}}^{A}} & \frac{\partial G_{k_{1}}^{I}}{\partial R_{k_{2}}^{A}} & \frac{\partial G_{k_{1}}^{I}}{\partial R_{k_{1}}^{I}} & \frac{\partial G_{k_{1}}^{I}}{\partial R_{k_{2}}^{I}}\\ \frac{\partial G_{k_{2}}^{I}}{\partial R_{k_{1}}^{A}} & \frac{\partial G_{k_{2}}^{I}}{\partial R_{k_{2}}^{A}} & \frac{\partial G_{k_{2}}^{I}}{\partial R_{k_{1}}^{I}} & \frac{\partial G_{k_{2}}^{I}}{\partial R_{k_{2}}^{I}} \end{array})|}_{\left(R_{k_{1}}^{A},R_{k_{2}}^{A},R_{k_{1}}^{I},R_{k_{2}}^{I}\right)=\left(0,0,0,0\right).}. \end{array}$$ (25)

By calculating the matrix components using Eqs (23)–(24), the linearized matrix *J*_0_ in Eq (25) is written as follows:

$$\begin{array}{c} J_{0}=(\begin{array}{cccc} qx_{11}L_{k_{1}}^{A} & qx_{12}L_{k_{2}}^{A} & px_{11}L_{k_{1}}^{A} & px_{12}L_{k_{2}}^{A}\\ qx_{21}L_{k_{1}}^{A} & qx_{22}L_{k_{2}}^{A} & px_{21}L_{k_{1}}^{A} & px_{22}L_{k_{2}}^{A}\\ qx_{11}L_{k_{1}}^{I} & qx_{12}L_{k_{2}}^{I} & px_{11}L_{k_{1}}^{I} & px_{12}L_{k_{2}}^{I}\\ qx_{21}L_{k_{1}}^{I} & qx_{22}L_{k_{2}}^{I} & px_{21}L_{k_{1}}^{I} & px_{22}L_{k_{2}}^{I} \end{array}), \end{array}$$ (26)

where

$$\begin{array}{ccc} & & x_{mn}=\frac{E(k_{m},k_{n})}{Q(k_{m})}\;\;\text{for}\;m,n=1,2, \end{array}$$ (27)

$$\begin{array}{ccc} & & L_{k_{m}}^{A}=\frac{Kk_{m}}{N}\cdot \frac{1}{\frac{Kk_{m}}{N}-a},\;L_{k_{m}}^{I}=\frac{Kk_{m}}{N}\cdot \frac{1}{\frac{Kk_{m}}{N}+b}\;\text{for}\;m=1,2. \end{array}$$ (28)

The eigenvalues *λ*_*j*_ (*j* = 1, 2, 3, 4) of *J*_0_ are obtained as follows:

$$\begin{array}{ccc} & & \lambda_{1}=\lambda_{2}=0,\;\;\lambda_{3}=\frac{-c_{1}+\sqrt{c_{1}^{2}-4c_{0}}}{2},\;\;\lambda_{4}=\frac{-c_{1}-\sqrt{c_{1}^{2}-4c_{0}}}{2}, \end{array}$$ (29)

where

$$\begin{array}{ccc} c_{0} & = & (x_{11}x_{22}-x_{12}x_{21})\left(qL_{k_{1}}^{A}+pL_{k_{1}}^{I}\right)\left(qL_{k_{2}}^{A}+pL_{k_{2}}^{I}\right), \end{array}$$ (30)

$$\begin{array}{ccc} c_{1} & = & -x_{11}\left(qL_{k_{1}}^{A}+pL_{k_{1}}^{I}\right)-x_{22}\left(qL_{k_{2}}^{A}+pL_{k_{2}}^{I}\right). \end{array}$$ (31)

The non-oscillatory equilibrium is stable if all the eigenvalues have absolute values less than unity. Otherwise the global oscillatory behavior is observed. We consider the condition for the phase transition between these two states. From *c*_1_ ≤ 0, the eigenvalue with the largest absolute value is *λ*_3_. The condition that |*λ*_3_| < 1 yields

$$\begin{array}{ccc} \sqrt{c_{1}^{2}-4c_{0}} & < & c_{1}+2. \end{array}$$ (32)

The above inequality requires (i) *c*_1_ + 2 > 0 and (ii) −*c*_0_ < *c*_1_ + 1. The condition (i) gives

$$\begin{array}{ccc} p & > & \frac{x_{11}L_{k_{1}}^{A}+x_{22}L_{k_{2}}^{A}-2}{x_{11}(L_{k_{1}}^{A}-L_{k_{1}}^{I})+x_{22}(L_{k_{2}}^{A}-L_{k_{2}}^{I})}. \end{array}$$ (33)

The condition (ii) gives

$$\begin{array}{ccc} h(p) & \equiv & \gamma_{2}p^{2}+\gamma_{1}p+\gamma_{0}>0, \end{array}$$ (34)

where

$$\begin{array}{ccc} \gamma_{0} & = & (x_{11}x_{22}-x_{12}x_{21})L_{k_{1}}^{A}L_{k_{2}}^{A}-x_{11}L_{k_{1}}^{A}-x_{22}L_{k_{2}}^{A}+1,\\ \gamma_{1} & = & (x_{11}x_{22}-x_{12}x_{21})\{L_{k_{1}}^{A}\left(L_{k_{2}}^{I}-L_{k_{2}}^{A}\right)+L_{k_{2}}^{A}\left(L_{k_{1}}^{I}-L_{k_{1}}^{A}\right)\}+x_{11}\left(L_{k_{1}}^{A}-L_{k_{1}}^{I}\right)+x_{22}\left(L_{k_{2}}^{A}-L_{k_{2}}^{I}\right),\\ \gamma_{2} & = & (x_{11}x_{22}-x_{12}x_{21})(L_{k_{1}}^{A}-L_{k_{1}}^{I})(L_{k_{2}}^{A}-L_{k_{2}}^{I}). \end{array}$$

Note that the sign of *γ*_2_ is the same as the sign of the assortative coefficient *r*, and therefore, *γ*_2_ ≠ 0 for correlated networks. Detailed calculations show that the sign of *h*(*p*) changes from negative to positive only once as *p* is varied from 0 to 1. Solving *h*(*p*) = 0, we obtain the critical fraction *p*_*c*_ for the phase transition as follows:

$$\begin{array}{ccc} p_{c}^{\text{bim}} & = & \frac{-\gamma_{1}+\sqrt{\gamma_{1}^{2}-4\gamma_{0}\gamma_{2}}}{2\gamma_{2}}. \end{array}$$ (35)

We can confirm that $p_{c}^{\text{bim}}$ satisfies the inequality condition (Eq 33). For uncorrelated networks with *r* = 0, *γ*_2_ = 0 and the critical fraction is reduced to

$$\begin{array}{ccc} p_{c}^{\text{bim}} & = & \frac{x_{11}L_{k_{1}}^{A}+x_{22}L_{k_{2}}^{A}-1}{x_{11}\left(L_{k_{1}}^{A}-L_{k_{1}}^{I}\right)+x_{22}\left(L_{k_{2}}^{A}-L_{k_{2}}^{I}\right)}, \end{array}$$ (36)

which is equivalent to that derived in the previous study [6].

#### Targeted inactivation

Next we consider the targeted inactivation. Without loss of generality, we can assume that the oscillator nodes with degree *k*_1_ are inactivated prior to those with degree *k*_2_. We separately treat the case where the phase transition occurs when the nodes with degree *k*_1_ are inactivated (Case 1) and the case where it occurs when the nodes with degree *k*_2_ are inactivated (Case 2).

**Case 1**: The phase transition occurs during the inactivation of the nodes with degree *k*_1_. All the oscillator nodes with degree *k*_2_ are active. Therefore, the Jacobian matrix corresponding to Eq (26) is reduced to

$$\begin{array}{cc} J_{0} & =(\begin{array}{ccc} x_{11}q_{1}L_{k_{1}}^{A} & x_{12}L_{k_{1}}^{A} & x_{11}p_{1}L_{k_{1}}^{A}\\ x_{21}q_{1}L_{k_{2}}^{A} & x_{22}L_{k_{2}}^{A} & x_{21}p_{1}L_{k_{2}}^{A}\\ x_{11}q_{1}L_{k_{1}}^{I} & x_{12}L_{k_{1}}^{I} & x_{11}p_{1}L_{k_{1}}^{I} \end{array}), \end{array}$$ (37)

where *p*_1_ is the proportion of the inactive oscillators to the total number of nodes with degree *k*_1_. The condition that all the eigenvalues have absolute values smaller than unity is given by

$$\begin{array}{ccc} p_{1} & > & p_{c}^{\left(k_{1}\right)}\equiv \frac{-(x_{11}x_{22}-x_{12}x_{21})L_{k_{1}}^{A}L_{k_{2}}^{A}+x_{11}L_{k_{1}}^{A}+x_{22}L_{k_{2}}^{A}-1}{\left(L_{k_{1}}^{A}-L_{k_{1}}^{I})\{-(x_{11}x_{22}-x_{12}x_{21})L_{k_{2}}^{A}+x_{11}\right\}}. \end{array}$$ (38)

By denoting the number of nodes with degree *k*_1_ by *N*_*k*_1__ and scaling the above fraction, we obtain the critical fraction as follows:

$$\begin{array}{ccc} p_{c}^{\text{bim}} & = & \frac{N_{k_{1}}}{N}p_{c}^{\left(k_{1}\right)}. \end{array}$$ (39)

**Case 2**: The phase transition occurs during inactivation of the nodes with degree *k*_2_ after all the nodes with degree *k*_1_ are inactivated. All the nodes with degree *k*_1_ are inactive. The proportion of the inactive oscillators with degree *k*_2_ to the total number of nodes with degree *k*_2_ is denoted by *p*_2_. A similar analysis to that in Case 1 gives the condition that all the eigenvalues have absolute values less than unity when

$$\begin{array}{ccc} p_{2} & > & p_{c}^{\left(k_{2}\right)}\equiv \frac{-(x_{11}x_{22}-x_{12}x_{21})L_{k_{1}}^{I}L_{k_{2}}^{A}+x_{11}L_{k_{1}}^{I}+x_{22}L_{k_{2}}^{A}-1}{(L_{k_{2}}^{A}-L_{k_{2}}^{I})\left\{-(x_{11}x_{22}-x_{12}x_{21})L_{k_{1}}^{I}+x_{22}\right\}}. \end{array}$$ (40)

By denoting the number of nodes with degree *k*_2_ by *N*_*k*_2__ and scaling the above fraction, we get the critical fraction as follows:

$$\begin{array}{ccc} p_{c}^{\text{bim}} & = & \frac{N_{k_{1}}}{N}+\frac{N_{k_{2}}}{N}p_{c}^{\left(k_{2}\right)}. \end{array}$$ (41)

#### Numerical validation

**Fig 6:**
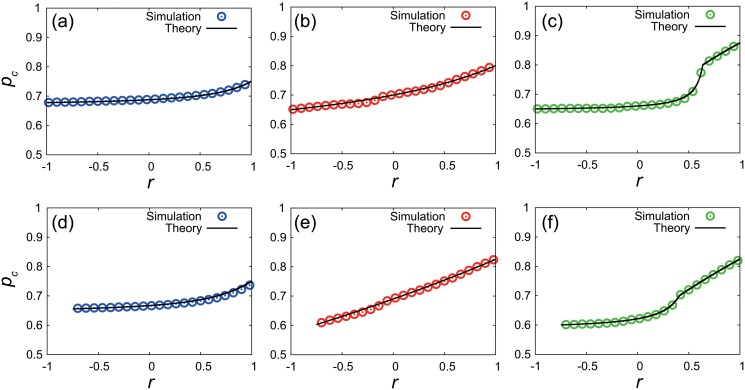
The critical value of *p*_*c*_ in correlated bimodal networks of coupled oscillators. The system size is *N* = 3000. (a)-(c) The results for networks where *k*_1_ = 600, *k*_2_ = 150, *N*_*k*_1__ = 600, and *N*_*k*_2__ = 2400: (a) random inactivation; (b) targeted inactivation of high-degree nodes; (c) targeted inactivation of low-degree nodes. (d)-(f) The results similar to (a)-(c), but for another set of networks where *k*_1_ = 450, *k*_2_ = 150, *N*_*k*_1__ = 900, and *N*_*k*_2__ = 2100.

The theoretical results for the critical fraction $p_{c}^{\text{bim}}$ in the bimodal networks are numerically validated. Fig 6 compares the critical fractions obtained by the theoretical analysis and those obtained by the numerical simulations. Figs 6(a)–6(c) show the results of the random inactivation, the targeted inactivation of high-degree nodes, the targeted inactivation of the low-degree nodes, respectively, for the network with *N* = 3000, *k*_1_ = 600, *k*_2_ = 150, *N*_*k*_1__ = 600, and *N*_*k*_2__ = 2400. The theoretical results are in good agreement with the numerical results in all the panels. The critical fraction monotonically increases with an increase in the assortativity coefficient *r* for all the inactivation types. The results show the positive role of assortativity in the dynamical robustness of bimodal networks. The discontinuity point found in Fig 6(c) corresponds to the boundary between Case 1 and Case 2, at which all the nodes with *k*_1_ have just become inactive. Figs 6(d)–6(f) show the results for another set of bimodal networks where *N* = 3000, *k*_1_ = 450, *k*_2_ = 150, *N*_*k*_1__ = 900, and *N*_*k*_2__ = 2100. The validity of our theoretical results are confirmed also in these networks.

## Discussion

We have studied the dynamical robustness of correlated networks consisting of diffusively coupled oscillators. The analyses of the dynamical robustness have been performed based on the critical point at which the global oscillatory dynamics is lost as the fraction of the inactive oscillators increases. To see the effect of the network assortativity, we have fixed the degree distribution of the network and changed the correlations of the degrees between the connected nodes using the two edge-rewiring methods. As a result, we have shown that the network assortativity enhances the dynamical robustness and the network disassortativity can have a positive or negative impact on the dynamical robustness depending on the edge-rewiring methods. We have investigated the similarity and difference between the networks generated by the edge-rewiring methods through the analyses of the oscillation amplitudes and the adjacency matrices representing the detailed network topology. We have found that the disassortative networks with the same assortativity coefficient can have qualitatively different types of topology, leading to the difference in the dynamical robustness. In the analyses of the correlated bimodal networks, we have theoretically derived the critical point as a measure of the dynamical robustness and confirmed the positive role of the network assortativity in the dynamical robustness. The previous studies have pointed out that the network assortativity is beneficial for the structural robustness of complex networks through the analyses of the percolation thresholds [12, 37, 45]. Therefore, we can conclude that the network assortativity improves the failure tolerance of complex networks from the viewpoints of both structural and dynamical robustness.

The numerical and theoretical analyses of the transition points in this study are expected to be applied to various real-world problems where dynamics is important, including how to effectively prevent epidemic spreading on transportation networks [46], how to stabilize electric power supply on power networks [47], and how to robustly keep neuronal firing activity on complex biological networks [48]. These real-world networks typically have degree correlations [8], and therefore, we need to take into consideration not only degree distributions but also degree correlations for characterizing the network structure. A future work is to understand the role of the network assortativity in the above phenomena through appropriate modeling of the node dynamics and detailed analyses of the actual network architectures.

## Supporting Information

S1 Fig Oscillation amplitudes in the networks with Poisson degree distributions, generated by the GER method. The model parameters are set at *N* = 1000 and *p* = 0.4. The node index is sorted in ascending order of the node degree. The top ((a)-(c)), middle ((d)-(f)), and bottom ((g)-(i)) panels correspond to random inactivation, targeted inactivation of high-degree oscillator nodes, and targeted inactivation of low-degree oscillator nodes, respectively. The left ((a), (d), (g)), center ((b), (e), (h)), and right ((c), (f), (i)) panels correspond to disassortative (*r* = −0.36), uncorrelated (*r* ≈ 0), and assortative (*r* = 0.36) networks, respectively.
(EPS)

S2 Fig Oscillation amplitudes in the networks with Poisson degree distributions, generated by the SER method. The model parameters are set at *N* = 1000 and *p* = 0.4. The node index is sorted in ascending order of the node degree. The top ((a)-(c)), middle ((d)-(f)), and bottom ((g)-(i)) panels correspond to random inactivation, targeted inactivation of high-degree oscillator nodes, and targeted inactivation of low-degree oscillator nodes, respectively. The left ((a), (d), (g)), center ((b), (e), (h)), and right ((c), (f), (i)) panels correspond to disassortative (*r* = −0.36), uncorrelated (*r* ≈ 0), and assortative (*r* = 0.36) networks, respectively.
(EPS)

S3 Fig Oscillation amplitudes in the networks with power-law degree distributions, generated by the GER method. The model parameters are set at *N* = 1000 and *p* = 0.5. The node index is sorted in ascending order of the node degree. The top ((a)-(c)), middle ((d)-(f)), and bottom ((g)-(i)) panels correspond to random inactivation, targeted inactivation of high-degree nodes, and targeted inactivation of low-degree nodes, respectively. The left ((a), (d), (g)), center ((b), (e), (h)), and right ((c), (f), (i)) panels correspond to disassortative (*r* = −0.48), uncorrelated (*r* ≈ 0), and assortative (*r* = 0.48) networks, respectively.
(EPS)

S4 Fig Oscillation amplitudes in the networks with power-law degree distributions, generated by the SER method. The model parameters are set at *N* = 1000 and *p* = 0.5. The node index is sorted in ascending order of the node degree. The top ((a)-(c)), middle ((d)-(f)), and bottom ((g)-(i)) panels correspond to random inactivation, targeted inactivation of high-degree nodes, and targeted inactivation of low-degree nodes, respectively. The left ((a), (d), (g)), center ((b), (e), (h)), and right ((c), (f), (i)) panels correspond to disassortative (*r* = −0.48), uncorrelated (*r* ≈ 0), and assortative (*r* = 0.48) networks, respectively.
(EPS)

S5 Fig The critical value *p*_*c*_ for correlated networks with system size *N* = 1500. In each panel, the numerically obtained values of the critical fraction *p*_*c*_ are plotted against the assortativity coefficient *r* for random and targeted inactivation. (a) Networks with Poisson degree distributions, generated by the GER method. (b) Networks with Poisson degree distributions, generated by the SER method. (c) Networks with power-law degree distributions, generated by the GER method. (d) Networks with power-law degree distributions, generated by the SER method.
(EPS)

S6 Fig The critical value *p*_*c*_ for correlated networks with coupling strength *K* = 40. In each panel, the numerically obtained values of the critical fraction *p*_*c*_ are plotted against the assortativity coefficient *r* for random and targeted inactivation. (a) Networks with Poisson degree distributions, generated by the GER method. (b) Networks with Poisson degree distributions, generated by the SER method. (c) Networks with power-law degree distributions, generated by the GER method. (d) Networks with power-law degree distributions, generated by the SER method.
(EPS)

S7 Fig The critical value *p*_*c*_ for correlated networks with parameter *b* = 5. In each panel, the numerically obtained values of the critical fraction *p*_*c*_ are plotted against the assortativity coefficient *r* for random and targeted inactivation. (a) Networks with Poisson degree distributions, generated by the GER method. (b) Networks with Poisson degree distributions, generated by the SER method. (c) Networks with power-law degree distributions, generated by the GER method. (d) Networks with power-law degree distributions, generated by the SER method.
(EPS)

S8 Fig The critical value *μ*_*c*_ for correlated networks of coupled heterogeneous oscillators with *δ* = 2. In each panel, the numerically obtained values of the critical point *μ*_*c*_ are plotted against the assortativity coefficient *r*. The parameter *α*_*i*_ (*i* = 1,…, *N*) is randomly chosen from a uniform distribution with the range of [*μ* − *δ*/2, *μ* + *δ*/2] where *μ* is the average of *α*_*i*_ and *δ* is the distribution width corresponding to the degree of heterogeneity. (a) Networks with Poisson degree distributions, generated by the GER method. (b) Networks with Poisson degree distributions, generated by the SER method. (c) Networks with power-law degree distributions, generated by the GER method. (d) Networks with power-law degree distributions, generated by the SER method.
(EPS)
